# Development and validation of a novel HILIC method for the quantification of low-levels of cuprizone in cuprizone-containing chow

**DOI:** 10.1038/s41598-021-97590-z

**Published:** 2021-09-09

**Authors:** Fengmei Zheng, Yiqing Lin, Pierre Boulas

**Affiliations:** Pharmaceutical Development, Biogen, 225 Binney St., Cambridge, MA 02142 USA

**Keywords:** Analytical chemistry, Demyelinating diseases

## Abstract

Cuprizone is an amide compound that has been wildly used in various animal studies, such as in the investigation of remyelination in mouse model. It is important to control the amount of cuprizone dosed in animals to be consistent as different amounts may lead to different clinical observations. Cuprizone is usually administrated as a minor component (i.e., 0.3%) of a mixture with powdered or pelleted rodent chow. Its low content, combined with the complex nature of chow, represents a significant challenge for the quantification of cuprizone in the mixture. To the best of our knowledge, no method has been reported in the literature so far. In this study, a simple, selective, and sensitive hydrophilic interaction liquid chromatographic method was developed for the quantification of cuprizone in cuprizone pre-clinical formulations. The analytical method comprises a fast ultrasound assisted extraction with acetonitrile/water as a solvent followed by gradient separation using a Waters Xbridge HILIC column with 0.1% TFA in water and acetonitrile as mobile phases and UV detection at 220 nm. The specificity, linearity, accuracy, repeatability, and limit of quantitation (LOQ) of the method were established. The method was determined to be linear in the range of 10–200 μg/mL. Accuracy was assessed by spiking a chow placebo with various amounts of a cuprizone reference standard to achieve target concentration levels and the recoveries were within the acceptance criterion of 90–110% of the target concentrations. Repeatability was demonstrated at the nominal concentration of 100 µg/mL and LOQ level of 2.5 μg/mL. This method has been demonstrated to be suitable for its intended use and has been successfully applied to the quantification of low levels of cuprizone in chow formulations. It was found that the cuprizone content in chow could varied significantly between batches and the potential causes of the variability were investigated.

## Introduction

**Figure 1:**
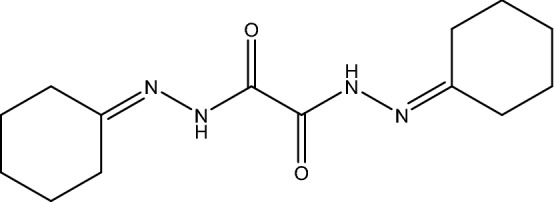
Chemical structure of cuprizone.

Cuprizone (oxalic acid bis(cyclohexylidene hydrazide)) (Fig. 1) is a well-known copper-cheating agent^1^. Cuprizone-induced toxicity has been extensively used to study experimental remyelination. In the cuprizone model, animals are fed with cuprizone to cause oligodendrocyte death and result in consistent demyelination^2–5^. The experimental results showed that different amount of the cuprizone might result in different clinical observations. For example, Carlton found that mice fed with different doses of cuprizone (ranging from 0.2 to 0.5%) mixed in basic chow showed signs of growth retardation in a dose-dependent manner^6^. In addition, Carlton and Ludwin observed the high mortality in mice administered with higher concentrations (0.5%) cuprizone in chow^6,7^; Zhen also found that mice from a 800 mg/kg dosing group died while mice from the 400 mg/kg dosing arm survived following 5 weeks of administrations^8^. Stidworthy et al. observed that 0.2% cuprizone was a more suitable dose than 0.4% in terms of mouse morbidity and weight loss^9^. Therefore, carefully controlling the amount of cuprizone in the cuprizone-based chows administrated to the animals is critical to achieve the desired results.

**Figure 2:**
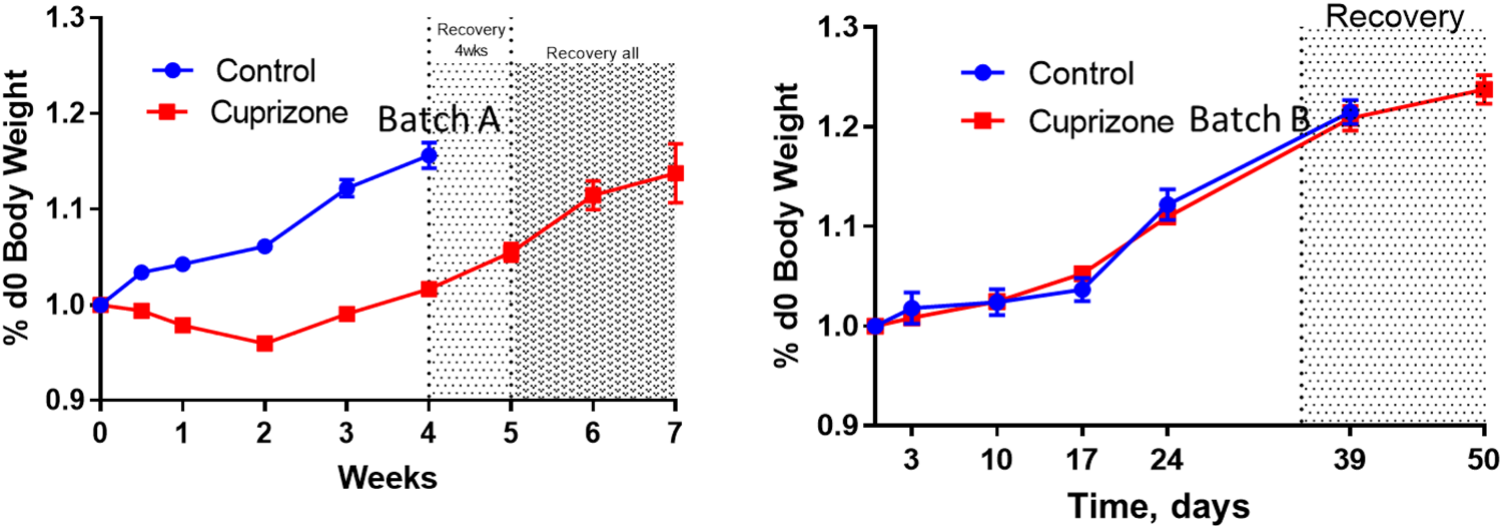
Weight loss of the mice were treated with chow placebo (control), 0.3% cuprizone-containing chow (batch A) and 0.3% cuprizone-containing chow (batch B).

**Figure 3:**
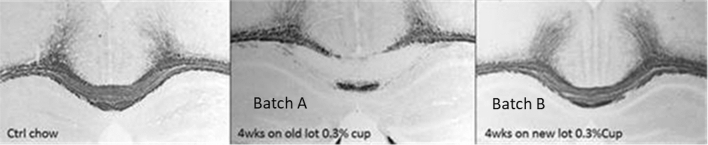
Demyelination detected in corpus callosum by black gold staining: the mouse on the left is on control chow, the middle mouse shows great demyelination of the corpus callosum using the batch A chow, and the mouse on the right was from the mouse using the batch B chow.

In our research work, cuprizone-containing chow was also used for demyelination studies with mouse model. Recently we found that when animals were fed with two different batches (batch A and batch B) of cuprizone-containing chow, the animal group fed with batch A showed significant demyelination of the corpus callosum with weight loss, which was a good indicator of a working model. However, the other animal group fed with batch B did not show the same pattern of weight loss. Histology analysis on mice fed with batch B of cuprizone chow confirmed that there was no demyelination (Figs. 2, 3).

Zhan et al. noted that cuprizone-containing pellets, instead of cuprizone in ground chow, failed to induce consistent demyelination^10,11^. To ensure reproducibility, Zhan’s lab had to prepare cuprizone-containing chow freshly every day by physically mixing cuprizone into ground rodent chow which was time consuming and a burden for the researchers. It was hypothesized that dosing of less than the target amount of cuprizone due to inhomogeneous mixing of cuprizone with chow or degradations of cuprizone during formulation preparation may account for the lack of demyelination effect observed in the study. To prove this hypothesis, a quantification method was required for the analysis of low levels of cuprizone in chow mixtures. To the best of our knowledge, no such method has been reported in the literature so far.

HPLC, with its high selectivity and accuracy, has been widely used for the quantification of analytes in complex samples^12^. In our lab, multiple attempts to develop a reversed phase HPLC method for the analysis of cuprizone in the chow were not successful due to complex components in chow placebo which resulted the coelution of cuprizone with the chow placebo peaks. In contrast to reversed phase HPLC, which employs a nonpolar stationary phase (SP) and a polar mobile phase (MP), hydrophilic interaction liquid chromatography (HILIC) uses a polar hydrophilic (normal) SP and an aqueous-polar organic solvent MP and provides a different elution order and selectivity from reversed phase HPLC^10^. In recent years, it has been increasingly applied to the separation and determination of polar pharmaceutical drugs and metabolites and provides a potential solution for the quantification of cuprizone in chow^13–17^.

In this study, a simple, selective, and sensitive HILIC method has been developed for the determination of low levels of cuprizone in cuprizone-based chows. Separation was achieved on a HILIC column using gradient elution with 0.1% TFA in water and acetonitrile as mobile phases and UV detection at 220 nm. The method was validated according to ICH guideline requirements and was determined to be linear in the range of 10–200 μg/mL. Method accuracy and recovery were assessed by spiking a chow placebo with various amounts of a cuprizone reference standard to achieve concentration levels of 10, 60, 100, 120 and 200 μg/mL (triplicate preparations). Method repeatability was demonstrated at the concentration of 100 µg/mL. The method was found to be specific with a quantitation limit of 2.5 μg/mL.

## Materials and methods

### Materials

Cuprizone was purchased from Sigma. HPLC grade Acetonitrile (MeCN), water, trifluoracetic acid (TFA) were purchased from Fisher Chemical (reagents are considered equivalent if performance as specified in system suitability is met). Cuprizone placebo sample (Global 16% Protein Chow) and Cuprizone Chow samples (0.3%wt of Cuprizone in protein chow) were provided by research group in Biogen (batches were manufactured for Biogen by Envigo, Frederick, MD, United States).

### Instrumentation

Agilent HPLC system (Agilent Technologies, Inc, Santa Clara, CA, United States or equivalent instrument) equipped with UV–vis absorbance detector and Empower 3 software (Waters Corporation, Milford, MA, United States) was employed for analyses. The analytical conditions were listed below (Table 1). Fisher Scientific FS30D sonicator (Waltham, MA, USA), METTLER TOLEDO analytical balance (Columbus, OH, USA) and Whatman 13 mm 0.45 µm GDX disposable filters (GE Healthcare Life Science, Marlborough, MA, USA) were used in sample preparations.

**Table 1:** Analytical conditions.

Instrument	Agilent 1200 HPLC			
Detector	UV 220 nm, bandwidth 4 nm, Reference off			
Software	Empower 3			
Mobile phase A (MPA)	0.1% TFA in water			
Mobile phase B (MPB)	Acetonitrile (MeCN)			
Diluent	50:50 (v/v) Acetonitrile: water			
Reference solution	Cuprizone in diluent			
Matrix reference solution/control solution	Cuprizone in placebo Chow blank			
Column	Waters Xbridge HILIC, 5 µm 4.6 × 250 mm			
Column temperature	25 °C			
Autosampler temperature	Ambient			
Injection volume	10 µL			
Flow rate	0.8 mL/min			
Retention time	~ 6.6 min			
Injections/sample	1			
Run time	14 min			
Calibration curve	y = Ax + B (not weighted)			
Elution mode	Gradient (see below)			
Time (min)	0	12	13	14
% MPA	5	50	5	5
% MPB	95	50	95	95

### Standard solution preparation

A stock solution (SS) of 800 µg/mL was prepared by dissolving 16 mg of cuprizone in 20 mL of 1:1 MeCN: water as the sample solvent. The working standards were prepared through a sequential dilution of the stock solution (SS) with the sample solvent as shown in Table 2. The standard solutions were stable at least for 7 days when stored at ambient temperature.

**Table 2:** Preparation of the working standard solutions.

Standard ID	SS (mL)	Final volume with diluent (mL)	Final concentration (μg/mL)
S-1	2.5	10	200
S-2	1.25	10	100
S-3	0.75	10	60
S-4	0.5	10	40
S-5	2.5 mL of S-6	10	10

A QC stock solution (QCS) of 200 µg/mL was prepared by dissolving 10 mg of cuprizone in 50 mL of the placebo blank diluent. The placebo blank diluent was prepared by adding ~ 4 g of chow placebo into 100 mL 1:1 MeCN: water, followed by vortexing and sonication for 5 min, and filtration with 0.45 μm membrane filter. The collected filtrate was used as a diluent for preparing the matrix reference standards. The QC samples of 10, 60, 100, 120 and 200 µg/mL were prepared in triplicate by diluting the QC stock solution sequentially with the placebo blank diluent according to Table 3.

**Table 3:** Preparation of QC samples.

Sample ID	QCS volume (mL)	Final volume with placebo diluent (mL)	Final TA concentration (μg/mL)
QC-1	10.0	10	200
QC-2	6.0	10	120
QC-3	5.0	10	100
QC-4	3.0	10	60
QC-5	0.5	10	10

The QC samples were only stable for 8 h when the solutions were stored at room temperature and about 16 h when stored in a refrigerator (5℃) which suggested that the samples should be freshly prepared for analysis.

### Sample preparation for HPLC analysis

Cuprizone chow and placebo chow samples were provided by our research group. For each sample, after grinding, around 2 g of fine powder was accurately weighed and transferred into a 50 mL of volumetric flask. About 40 mL of sample solvent then was added to the flask. To increase extraction efficiency, the sample was ultra-sonicated for 5 min and then QS with the sample solvent to 50 mL. 5 mL of the resulting suspensions was then filtrated through a 0.45 µm membrane filter. The first 3 mL (1 mL each) and the rest of the filtrate were collected for HPLC analysis respectively. The results shown 98–102% recovery was achieved after discarding the first 3.0 mL of filtrate.

### Method validation

The method was validated in accordance with ICH Q2(R1) for specificity, linearity and range, repeatability, and accuracy. Filter study and the solution stability were also investigated.

## Results and discussion

### HILIC method development and optimization

**Figure 4:**
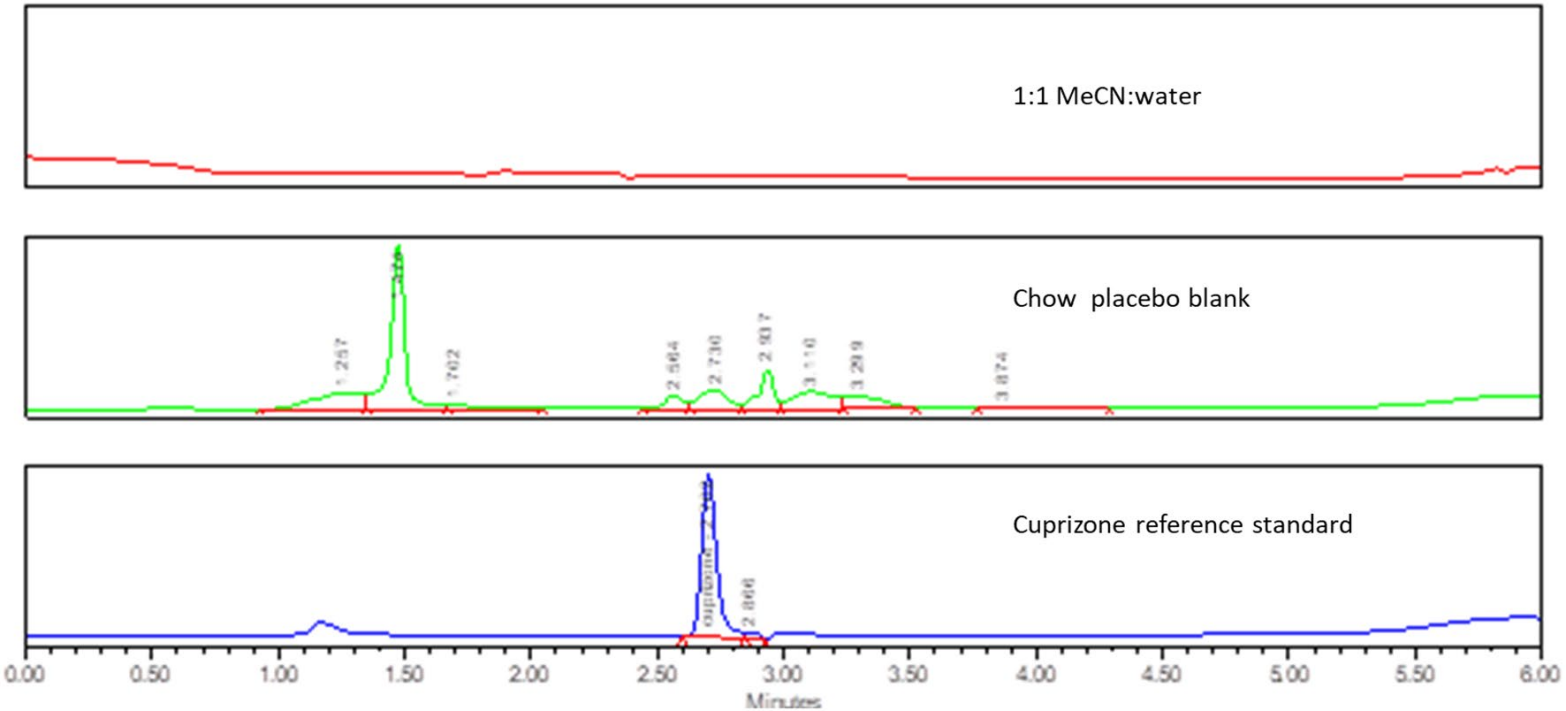
HPLC chromatograms of cuprizone samples separated with Altantis T3 column.

**Figure 5:**
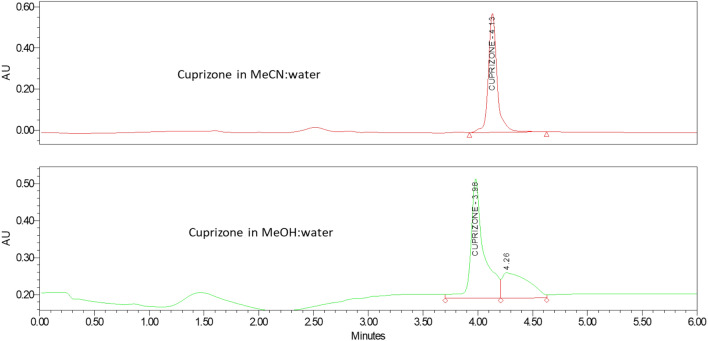
HPLC chromatograms for cuprizone reference standards, Xbridge HILIC column, (top) 1:1 MeCN: water as sample solvent; (bottom) 1:1 MeOH: water as sample solvent.

Cuprizone contains both hydrophobic and hydrophilic functional groups and thus could theoretically be analyzed by either RP-HPLC or HILIC-HPLC^18,19^. Both methods were explored to achieve the retention of cuprizone on column and the separation of cuprizone from the chow placebo interference peaks. Multiple RP-HPLC columns were screened and in all the cases cuprizone was observed to either elute with the solvent front or coelute with the chow placebo peaks, such as using an Atlantis T3 column as shown in Fig. 4. In comparison, cuprizone was separated from the chow placebo peaks when using a Waters Xbridge HILIC column (Fig. 5). Using this column, mobile phase and sample diluent were then optimized.

#### Column selection

Table 4 summarized the experimental results from screening of multiple RP and HILIC columns to achieve the separation of cuprizone from the chow placebo interference peaks. The results indicated that none of the three RP columns were suitable as retention of cuprizone on the column or separation of cuprizone from the interference peaks was not achievable. Of the two HILIC columns screened, the Waters Xbridge HILIC column (5 μm, 4.6 × 250 mm) showed promising results and therefore was selected for further investigation.

**Table 4:** Summary of the columns screened for the method development.

Column tested	Column type	Results
Agilent Eclipse XDB-C18	RP	Cuprizone elute with solvent front
Phenomenex Kinetex-C18	RP	Cuprizone elute with solvent front
Atlantis T3	RP	Cuprizone co-eluted with chow placebo peaks
Atlantis HILIC Silica	HILIC	Cuprizone co-eluted with chow placebo peaks
Waters Xbridge HILIC, 5 μm 4.6 × 250 mm	HILIC	Cuprizone retention on column and separation from interference peaks achieved

#### Effect of sample solvent

It was found that sample solvent had an impact on the cuprizone peak shape. As indicated in Fig. 5, when 1:1 mixture of MeOH/water was used as the sample solvent, peak splitting was observed. In contrast, decent peak shape was achieved when using 1:1 mixture of MeCN/water as the sample solvent. The observed distortion of peak shape may arise from mismatch of sample solvent and mobile phase, which is one of the most common challenges in HILIC. Compared to 1:1 MeCN/water, 1:1 MeOH/water has higher elution strength in HILIC, which impairs the partitioning of the analytes into the stationary phase and results in peak distortion.

#### Effect of mobile phase

**Figure 6:**
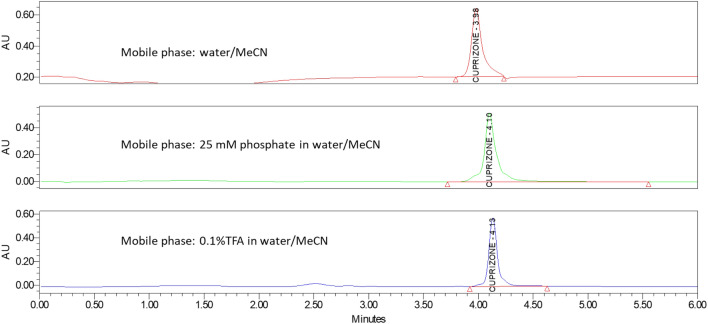
HPLC chromatogram of Cuprizone in Xbridge HILIC column with different mobile phases (from top to bottom): water/ MeCN; 25 mM phosphate in water/ MeCN and 0.1%TFA in water/ MeCN.

Mobile-phase pH and buffer ions play an important role in HILIC retention since they can influence the electric charge state of both ionizable solutes and stationary phase, which may affect the thickness of the stagnant enriched aqueous layer on the surface of the stationary phase. This is turn can lead to an additional ionic interaction which can impact the solutes retention. To examine this effect (analyte retention and peak shape), three mobile phases: water/MeCN, 0.1%TFA in water/MeCN and 25 mM phosphate buffer/MeCN were investigated (Fig. 6). The results indicated that pH and ion strength did not significantly affect the retention of cuprizone on the Waters Xbridge HILIC column. 0.1% TFA in water/MeCN was eventually selected as the mobile phases for better peak retention and peak shape.

**Figure 7:**
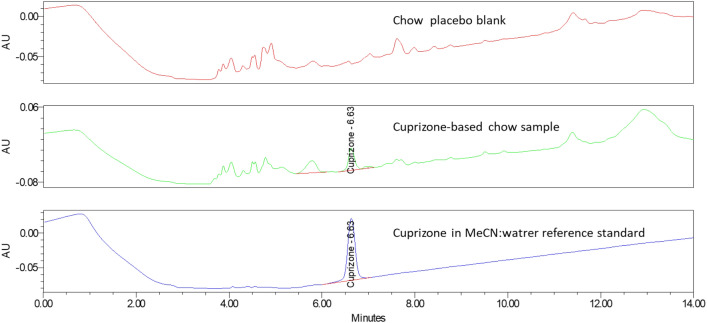
HPLC chromatogram of cuprizone samples in Xbridge HILIC column.

Through optimization, the analytical method conditions listed in Table 1 were developed for further validation. Representative chromatograms are shown in Fig. 7.

### Method validation

**Figure 8:**
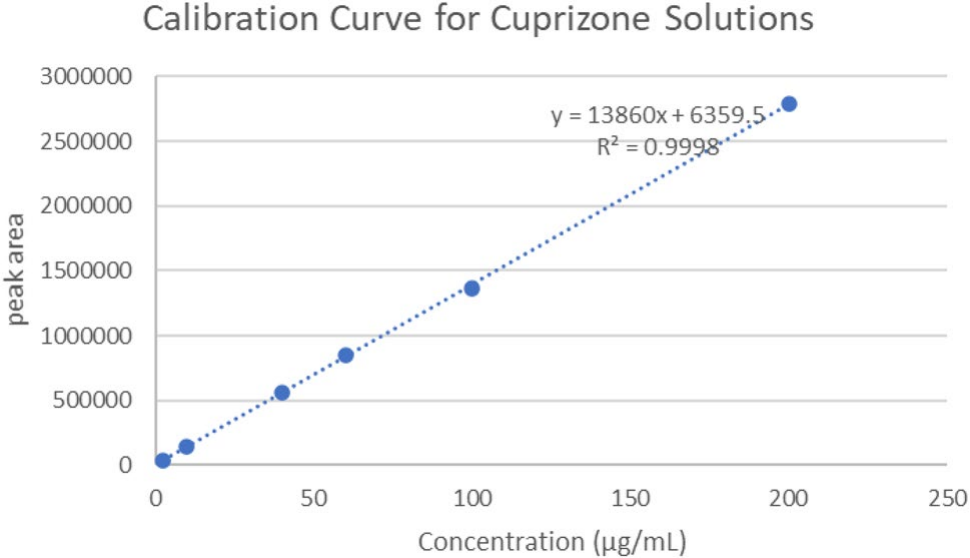
Calibration curve for cuprizone solutions.

The calibration curve for cuprizone was obtained using a series of standard solutions over the concentration range of 10–200 µg/mL. A linear relationship between the peak area of the cuprizone and the concentration of the standard with R^2^ = 0.9998 was obtained (Table 5, Fig. 8). The percent recovery for each standard prepared met the acceptance criterion of 90–110% of the nominal concentration. The correlation coefficient of the calibration curve met the acceptance criterion of R^2^ ≥ 0.99.

**Table 5:** Results of solvent standards for validation.

Solvent standard ID	TA conc. (μg/mL)	TA peak area	Calc. conc. (μg/mL)	%Recovery
S-0 (solvent blank)	0	NA	NA	NA
S-6	2.50	36,627	2.67	107.0
S-5	10	146,507	10.8	107.9
S-4	40	566,680	41.7	104.4
S-3	60	851,289	62.7	104.5
S-2	100	1,369,820	100.0	100.0
S-1	200	2,784,301	205.1	102.5
Correlation coefficient			0.9998	
Slope			13,843	
Intercept			8553.2

The analytical accuracy and recovery of the method was assessed using 9 determinations over 5 concentration levels (3 replicates/concentration level) covering the specified range of 10–200 µg/mL. The QC samples were prepared by adding reference standard to the placebo blank matrix.

The method was shown to be accurate at concentration levels of 10, 60, 100, 120 and 200 μg/mL with RSD values (triplicate preparations) of 0.51%, 0.75%, 0.47%, 0.28% and 0.72% for cuprizone, respectively. The percent recovery for each standard prepared met the acceptance criterion of 90–110% of the nominal concentration (Table 6).

**Table 6:** Accuracy and of recovery of the cuprizone method.

Sample solution ID	Concentration (μg/mL)	Recovery concentration (μg/mL)	Recovery (%)	RSD (n = 3)
QC-1	200	198.5	99.3	0.72
QC-2	120	112.5	93.7	0.28
QC-3	100	101.3	101.3	0.47
QC-4	60	61.5	102.5	0.75
QC-5	10	10.5	104.9	0.51

The repeatability of the method was determined by six injections of the 100 µg/mL standard at the beginning of the analysis. Cuprizone peak area and Cuprizone retention time were evaluated. All acceptance criteria were met. The results are shown in Table 7.

**Table 7:** The repeatability for the method validation (100 µg/mL reference standard).

	Cuprizone peak area	Cuprizone retention time (min)
Injection 1	1,369,820	6.64
Injection 2	1,373,874	6.62
Injection 3	1,373,711	6.62
Injection 4	1,372,307	6.62
Injection 5	1,373,168	6.62
Injection 6	1,365,070	6.60
Average	1,372,576	6.62
% RSD	0.12	0.14
Acceptance criterion ≤ % RSD	2	5

**Figure 9:**
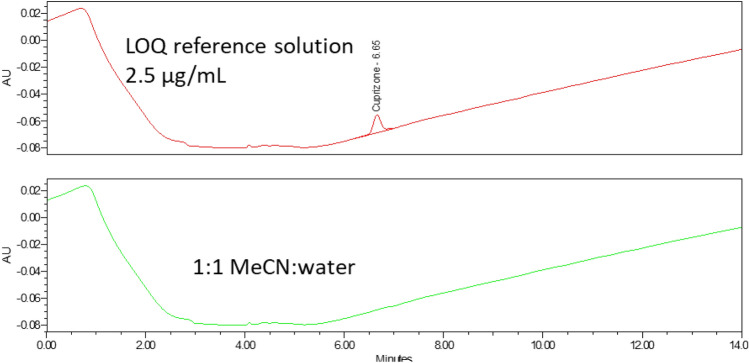
Chromatogram of LOQ reference standard (2.5 μg/mL) and sample solvent (1:1 MeCN: water).

The limit of quantification (LOQ) was 2.5 µg/mL, determined as the concentration of cuprizone that gives rise to peak height with a S/N ≥ 10 (Fig. 9).

System stability/reproducibility of the standards on the autosampler for the duration of the analytical run was evaluated by comparison of the average of the six system suitability injections at the beginning of the analysis with the injection of 100 µg/mL at the end of the analysis. The peak areas obtained met the acceptance criterion of < 5% change over the course of the analysis (Table 8).

**Table 8:** Autosampler stability for validation.

	Cuprizone peak area	%Difference
System suitability (average)	1,372,576	+ 0.09%
End of analysis	1,373,874	

All acceptance criteria were met and the analytical method for the determination of Cuprizone in Cuprizone containing chow was validated.

### Batch analysis of cuprizone-containing chow

The HILIC-HPLC method was applied to the determination of cuprizone content in the two batches of chow (batch A and B) used in the animal studies. As summarized in Table 9, compared to batch A with a cuprizone content of 0.32%, batch B had a much lower cuprizone content of 0.08%. This explains why demyelination and weight loss were not observed in the animal group fed with batch B.

**Table 9:** Cuprizone amount in the cuprizone-containing chow batches.

Sample information	Label claim (%)	%Cuprizone detected with the HILIC HPLC method	%Label claim
0.3% cuprizone chow (batch A)	0.3	0.32	107
0.3% cuprizone chow (batch B)	0.3	0.08	27

### Investigation of batch-to-batch variability of cuprizone content in chow formulation

Cuprizone-containing chow is typically made by mixing cuprizone and chow together with the addition of water at the end of the mixing to generate pellets followed by drying the pellets at 50 °C under vacuum for a few hours. Three potential causes, independently or together, could account for the low cuprizone content in cuprizone-containing chow batch B: (1) inhomogeneous mixing of cuprizone with chow; (2) degradation of cuprizone during mixing and/or drying; (3) degradation of cuprizone during storage of chow before use. Since it was difficult to know if inhomogeneous mixing was a cause for the low chow content and the chow formulation was demonstrated to be stable under typical storage conditions, we focused our studies on exploring the potential degradation of cuprizone during mixing and/or drying.

To determine if the water amount and/or the drying conditions have any impact on the degradation of cuprizone, samples were prepared by mixing cuprizone with the chow placebo and adding different amount of water to the mixture. The samples were then stored at ambient temperature or dried at 50 °C a vacuum oven for different duration. After drying, the samples were analyzed using the developed HILIC-HPLC method. The experimental design and results were summarized in Table 10.

**Table 10:** Stability of cuprizone in different conditions.

Sample information	Stressed condition	Observation
Pure cuprizone	Vacuum at 50 ℃ for 24 h	Stable with negligible degradation
Pure cuprizone + 18% water	Vacuum at 50 ℃ for 24 h	Stable with negligible degradation
0.3% cuprizone + chow placebo + 18% water	Stored at room temperature for 24 h followed by vacuum at 50 ℃ for 6 h (standard) or 24 h (stressed)	10% degradations were observed in all samples regardless of drying conditions
0.3% cuprizone + chow placebo + 30% water		40% degradation were observed in all samples regardless of drying duration
0.3% cuprizone + chow placebo + 50% water		60% degradation were observed in all samples regardless of drying duration

As shown in Table 10, when mixed only with water, cuprizone was stable even subject to high drying temperature of 50 °C for 24 h. On the contrary, when mixed with both chow and water, significant degradation of cuprizone was observed. The drying duration seemed to have no impact on the degree of degradation. In addition, higher water content resulted in more degradation. Therefore, it appears that cuprizone can react with components in chow in the presence of water. This might partially account for the low cuprizone content observed in batch B.

## Conclusions

In this study, a novel, sensitive, and selective HILIC method for the determination of cuprizone has been developed. The method was validated according to ICH Q2(R1). No placebo matrix component was found to interfere the cuprizone determination. Good linearity and sensitivity were obtained as well. The method was successfully applied to the determination of low level of cuprizone in chow. In addition, it was found that cuprizone could react with other components in chow in the presence of water, which might partially account for the observed chow batch-to-batch variability in cuprizone content.
